# EEG-Based Seizure Prediction Approaches Within Clinically Relevant Pre-seizure Windows Using Scalp EEG Datasets: A Systematic Review

**DOI:** 10.7759/cureus.109989

**Published:** 2026-05-31

**Authors:** Aesha Ali Alasade, Ahmed-Lamin Gehani, Hind Mohammedsalih Osman, Abdulwahhab M Al-Shaikhli, Abdelrahman Idris Mohamed Idris, Asim Ahmed, Almalik Abla

**Affiliations:** 1 Medicine, University of Jordan, Amman, JOR; 2 General Practice, Near East University, Lefkosa, CYP; 3 Internal Medicine, Ministry of Health, Sultanate of Oman, Al Buraimi, OMN; 4 Neurology, Gardenia Medical Center, Doha, QAT; 5 Internal Medicine, Kassala Teaching Hospital, Kassala, SDN; 6 Medicine and Surgery, University of Gezira, Wad Madani, SDN; 7 Neurology, Alshaya Medical Complex, Ar Rass, SAU

**Keywords:** false alarm rate (false predictions per hour), machine learning, preictal window, scalp electroencephalography (eeg), seizure prediction

## Abstract

This systematic review synthesized evidence on EEG-based seizure prediction within clinically relevant pre-seizure windows, focusing on deep learning and machine learning models evaluated on public datasets. The review aimed to summarize predictive performance, including sensitivity, receiver operating characteristic curve findings when reported, and false-alarm outcomes for scalp EEG seizure prediction systems. It also examined methodological heterogeneity across studies, including the preictal window, defined as the period before seizure onset used for prediction; the seizure prediction horizon, defined as the minimum warning interval before seizure onset; and the seizure occurrence period, defined as the interval during which a seizure is expected after an alarm. Additional objectives were to compare validation strategies, including patient-wise generalization, and to identify design features most consistently associated with clinically actionable performance. The included evidence showed substantial variability in reported performance. It was limited by heterogeneous study designs, frequent reliance on patient-specific internal validation, limited external validation, inconsistent reporting of false alarms, and high or unclear risk of bias. Although scalp EEG seizure prediction models have shown potential to achieve moderate-to-high sensitivity in selected settings, confidence in their real-world generalizability remains limited. Overall, the findings suggest that clinical translation will require more standardized prediction windows, clearer reporting of alarm burdens, stronger validation frameworks, and prospective evaluations under real-world conditions.

## Introduction and background

Epilepsy is a chronic neurological disorder characterized by an enduring predisposition to generate epileptic seizures and by associated neurological, cognitive, psychological, and social consequences [1,2]. Despite significant advances in diagnosis and treatment, epilepsy continues to impose substantial global morbidity, with ongoing risks of injury, psychosocial restriction, and reduced quality of life among affected individuals [1,2]. Although antiseizure medications achieve seizure freedom for many patients, a clinically significant subgroup continues to experience seizures despite appropriate therapy, and the concept of drug-resistant epilepsy has been formalized to guide evaluation and escalation of care [3,4]. For these individuals, unpredictable seizures remain a central driver of harm, limiting independence and complicating education, employment, and daily activities.

Several technical terms are central to this review. Interictal refers to the period between seizures, whereas preictal refers to the period before seizure onset during which EEG changes may indicate increased seizure likelihood. Seizure detection identifies an ongoing or already occurring seizure, seizure prediction aims to provide a warning before an imminent seizure within a defined pre-seizure interval, and seizure forecasting estimates a broader future period of increased seizure risk. The seizure prediction horizon refers to the minimum interval between an alarm and seizure onset. In contrast, the seizure occurrence period is the time window during which a seizure is expected following an alarm. False-alarm burden describes the frequency and practical impact of alarms not followed by seizures, and time in warning refers to the proportion of monitoring time during which the system remains in an alarm or warning state.

Anticipating seizures, rather than reacting to them, is therefore a clinically meaningful target. Foundational work has outlined the conceptual basis of seizure prediction, including the distinction between interictal and preictal brain states, the importance of patient-specific modeling, and the practical constraints imposed by false alarms and heterogeneous seizure dynamics [5]. Translational studies have provided proof of concept that seizure risk can be estimated over time in selected populations, including long-term monitoring and advisory approaches, while also demonstrating challenges in maintaining reliability across individuals and contexts [6]. Reviews of therapeutic and assistive devices have further emphasized that real-world utility depends not only on sensitivity but also on an acceptably low false-alarm burden, clearly defined operating horizons, and feasibility of continuous deployment [7]. These challenges are clinically important because frequent false alarms may increase anxiety, reduce trust in warning systems, and limit long-term usability. At the same time, data leakage or weak validation may lead to an overestimation of model performance.

Over the last two decades, progress in seizure prediction research has been accelerated by shared EEG resources and open benchmarking. PhysioNet has supported broad access to physiologic datasets and reproducible evaluation, enabling iterative methodological development across research groups [8]. Within this ecosystem, the CHB MIT Scalp EEG Database has been widely used to develop and compare scalp EEG algorithms in a standardized format, supporting investigations that are closer to noninvasive clinical workflows than intracranial-only approaches [9]. Additional initiatives, such as the EPILEPSIAE database, have expanded access to curated EEG recordings and metadata, facilitating cross-study comparisons and, where available, multicenter model development [10]. Community challenges have catalyzed methodological innovation by providing shared data and evaluation goals, including competitions that aim to advance false-alarm reduction while preserving clinically meaningful sensitivity [11].

Alongside these data resources, deep learning and other modern machine learning methods have increasingly been applied to seizure prediction, motivated by their ability to learn hierarchical representations from complex time series data. These approaches are especially relevant because EEG signals contain temporal, spectral, and spatial patterns that may be difficult to characterize using manual feature interpretation alone. Machine learning in this review refers broadly to computational prediction models, including conventional feature-based classifiers and deep learning architectures such as convolutional, recurrent, graph-based, and sequence learning models. Proof-of-concept studies have described fully automated, patient-tunable systems and explored pathways toward low-power or mobile deployment, thereby supporting the feasibility of near-real-time decision support in constrained computing environments [12]. Nevertheless, dataset-focused reviews have highlighted persistent problems in the literature, including conflation of detection, prediction, and forecasting; inconsistent patient-wise separation; and methodological heterogeneity that limits fair comparison across studies and databases [13]. Performance syntheses further caution that choices around the seizure prediction horizon and the definitions of seizure occurrence periods, post-processing, alarm counting, and validation design can materially influence reported accuracy and generalizability, increasing the risk of overly optimistic conclusions when evaluation is not clinically aligned [14].

Accordingly, this systematic review aimed to synthesize evidence on machine learning and deep learning approaches for EEG-based seizure prediction within clinically relevant pre-seizure windows, defined here as pre-seizure intervals that are early enough to allow warning or protective action while remaining close enough to seizure occurrence to support clinically meaningful alarm performance, with a focus on models evaluated on public datasets. The primary objective was to summarize the predictive performance of seizure prediction systems using scalp EEG recordings, including sensitivity, area under the receiver operating characteristic curve, and false-alarm metrics. Secondary objectives were to describe methodological heterogeneity, including preictal window definitions, seizure prediction horizons, post-processing, and alarm handling; to compare validation strategies, including patient-wise generalization; and to identify design features most consistently associated with clinically usable performance.

## Review

Methods

Reporting Framework and Review Design

This systematic review was designed to evaluate whether public scalp EEG datasets support clinically meaningful seizure early warning within short- to intermediate-pre-seizure windows, with particular attention to 5- to 30-minute horizons, while also considering longer, explicitly defined prediction windows when they addressed pre-seizure warning rather than seizure detection alone. The review focused on machine learning and deep learning approaches and was written in accordance with Preferred Reporting Items for Systematic Reviews and Meta-Analyses (PRISMA) 2020 reporting guidance [15]. The review protocol was not prospectively registered.

Eligibility Criteria

Eligibility criteria were defined prior to screening using the PICOS framework (Table 1). We included primary studies published between January 2016 and December 2025 that analyzed human scalp EEG, used public or open datasets such as the CHB MIT database or comparable sources, addressed seizure prediction or early warning prior to seizure onset rather than detection only, and reported at least one usable performance outcome, including sensitivity or recall, area under the receiver operating characteristic curve, and false-alarm metrics such as false alarms per hour. For this review, seizure prediction or early warning was defined as a model task that attempted to identify a preictal state and provide a risk estimate or warning before seizure onset, rather than simply detecting an ongoing or already occurring seizure. Machine learning was defined broadly as computational prediction modeling, including feature-based supervised learning methods and deep learning architectures. Studies that did not use machine learning or deep learning approaches were excluded because they were outside the scope of the review. The 5- to 30-minute range was treated as the main clinically relevant reference window rather than an absolute exclusion threshold. Studies using longer explicit preictal windows or prediction horizons were retained only when their task remained pre-seizure prediction or early warning and when extractable performance outcomes were available. We excluded reviews, protocols, editorials, non-human studies, studies without usable quantitative outcomes, and studies whose task definition did not match prediction or early warning.

**Table 1:** PICOS eligibility framework for study inclusion PICOS: population, intervention, comparator, outcomes, and study design, AUROC: area under the receiver operating characteristic curve, EEG: electroencephalography

Component	Specification
Population or data (P)	Human scalp EEG recordings, noninvasive, from public or open datasets.
Intervention or index (I)	Seizure prediction or early warning models applied to scalp EEG using machine learning and/or deep learning approaches.
Comparator (C)	Alternative models or baselines were reported; a comparator was not required for inclusion.
Outcomes (O)	Sensitivity or recall, area under the receiver operating characteristic curve when reported, and false-alarm reporting, such as false alarms per hour.
Study type (S)	Primary model development and validation studies, retrospective and/or prospective.

Information Sources and Search Strategy

The final database search was conducted on January 10, 2026, and eligible studies were limited to primary research articles published between January 2016 and December 2025. We searched multiple bibliographic databases selected to capture both clinical and engineering literature, including PubMed/MEDLINE, IEEE Xplore, Scopus, and Web of Science. Searches used structured Boolean combinations of epilepsy or seizure terms with prediction, forecasting, or early warning concepts, EEG terms, public or scalp dataset terms, and machine learning or deep learning terms. These machine learning and deep learning terms were included because the review specifically aimed to evaluate computational EEG-based seizure prediction models rather than all seizure-monitoring, seizure-detection, or non-computational prediction approaches. Searches were limited to English-language records. This English language restriction is acknowledged as a limitation because relevant non-English studies may have been missed. Full database-specific search strategies, including exact search strings, filters, and date limits, are provided in the Appendices. The databases, core concepts, and search limits are summarized in Table 2.

**Table 2:** Database search strategy and limits CHB MIT: Children’s Hospital Boston Massachusetts Institute of Technology scalp EEG database, CNN: convolutional neural network, EEG: electroencephalography, LSTM: long short-term memory

Item	Details
Databases searched	PubMed or MEDLINE, IEEE Xplore, Scopus, and Web of Science.
Core concepts	Epilepsy or seizures; prediction, forecasting, or early warning; EEG or electroencephalography; machine learning or deep learning architectures; scalp EEG or public datasets.
Example search string	(epilepsy OR seizure) AND (prediction OR forecast OR forecasting OR early warning) AND (EEG OR electroencephalography) AND (machine learning OR deep learning OR CNN OR LSTM OR transformer) AND (scalp OR public dataset OR CHB MIT).
Limits or filters	English language records; eligible publication period from January 2016 to December 2025; final search conducted on January 10, 2026.
Full search strategies	Database-specific strings are provided in the Appendices.

Study Selection Process

All retrieved records were exported to Zotero for reference management and duplicate identification, and screening decisions were tracked in Microsoft Excel. Screening was performed in two stages by two reviewers. First, titles and abstracts were screened to exclude clearly irrelevant records. Second, full-text articles were assessed against the eligibility criteria. Disagreements at either stage were resolved by discussion and consensus between the two reviewers; no third reviewer was used. Because this review used published study reports rather than patient-level records, de-identification of individual records was not applicable. Full-text articles that could not be accessed after reasonable retrieval attempts via journal websites, DOI links, institutional access, and open repositories were excluded at the full-text stage and recorded with the relevant reason for exclusion. A summary of the study selection, including the number of records retrieved, full-text articles assessed, full-text exclusions, inaccessible full texts, and final included studies, is presented in the Results section and PRISMA flow diagram.

Data Extraction

A standardized extraction form was used to ensure consistent capture of study, dataset, and model characteristics and to support cross-study comparability. The standardized extraction form is provided in the Appendices. Extracted items included dataset(s) used, population or sample size, and seizure counts when reported; EEG acquisition details including channels, montage, and sampling rate; definitions of interictal and preictal windows, seizure prediction horizon, and seizure occurrence period when specified; preprocessing; input representations such as raw EEG, spectrogram or time-frequency features, wavelet bands, and synchronization features; model family such as convolutional neural network (CNN), long short-term memory (LSTM), graph convolutional network (GCNs), or feature-based machine learning; validation design including patient-specific splits, leave one patient out, leave one seizure out, chronological testing, and external validation; and performance outcomes. When false-alarm outcomes were reported using different definitions or units, we extracted the metric exactly as stated by the authors and recorded its unit and calculation method to avoid inappropriate conversions.

Outcomes

The prespecified primary outcomes were sensitivity or recall and false-alarm burden, preferably false alarms per hour or an equivalent alarm rate. The area under the receiver operating characteristic curve was extracted when reported. Secondary outcomes included specificity, accuracy, warning or anticipation time, time in warning, and authors’ feasibility claims.

Risk of Bias Assessment

Risk of bias was assessed using a simplified PROBAST-style framework adapted for EEG-based seizure prediction model studies [16]. This adapted approach was used because the included studies were heterogeneous computational prediction studies, and many did not report all elements required for direct application of the full standard PROBAST tool. Each study was rated low, high, or unclear across four domains. Domain 1 was participants or data selection. Domain 2 was predictors or EEG handling, including leakage risk related to preprocessing, segmentation, seizure overlap, and separation of training and testing data. Domain 3 was outcome definition, including seizure onset labeling, preictal window clarity, seizure prediction horizon, and seizure occurrence period. Domain 4 was analysis and validation, including generalization testing, patient-wise separation, temporal separation, external validation, and completeness of reporting. Overall risk of bias was rated high if any domain was high, particularly when leakage risk or validation concerns were present. Overall risk of bias was rated low if all domains were low, allowing at most one unclear judgment. Overall risk of bias was rated as unclear because reporting limitations prevented a confident overall judgment [16]. Studies judged to be at high risk of bias were not excluded from the qualitative synthesis, but their findings were interpreted with caution regarding reliability, generalizability, and clinical translation.

Synthesis Approach

A narrative synthesis was performed because quantitative pooling was not appropriate. The included studies showed substantial methodological and clinical heterogeneity in datasets, EEG sources, preictal definitions, seizure prediction horizons, seizure occurrence periods, model architectures, preprocessing methods, validation strategies, post-processing rules, and false-alarm definitions. Because these differences prevented reliable harmonization of outcomes, no meta-analysis, meta-regression, pooled effect estimate, p-value synthesis, or pooled confidence interval calculation was performed.

Findings were synthesized descriptively by grouping studies according to dataset type, EEG source, prediction horizon, seizure occurrence period, model family, validation design, and completeness of alarm-related reporting. Particular attention was given to sensitivity or recall, false alarms per hour, time in warning when reported, and whether validation was patient-specific, chronological, leave-one-out, independent, or externally validated. Performance metrics were extracted and reported as described in the original study, and direct numerical comparisons across studies were avoided because definitions and validation designs were not sufficiently comparable.

This narrative approach was used to prioritize clinically meaningful interpretation of seizure prediction performance, with greater caution applied to studies with high or unclear risk of bias, patient-specific internal validation, incomplete alarm reporting, unclear train-test separation, possible overlapping window leakage, or limited external validation.

Results

Study Selection

**Figure 1:**
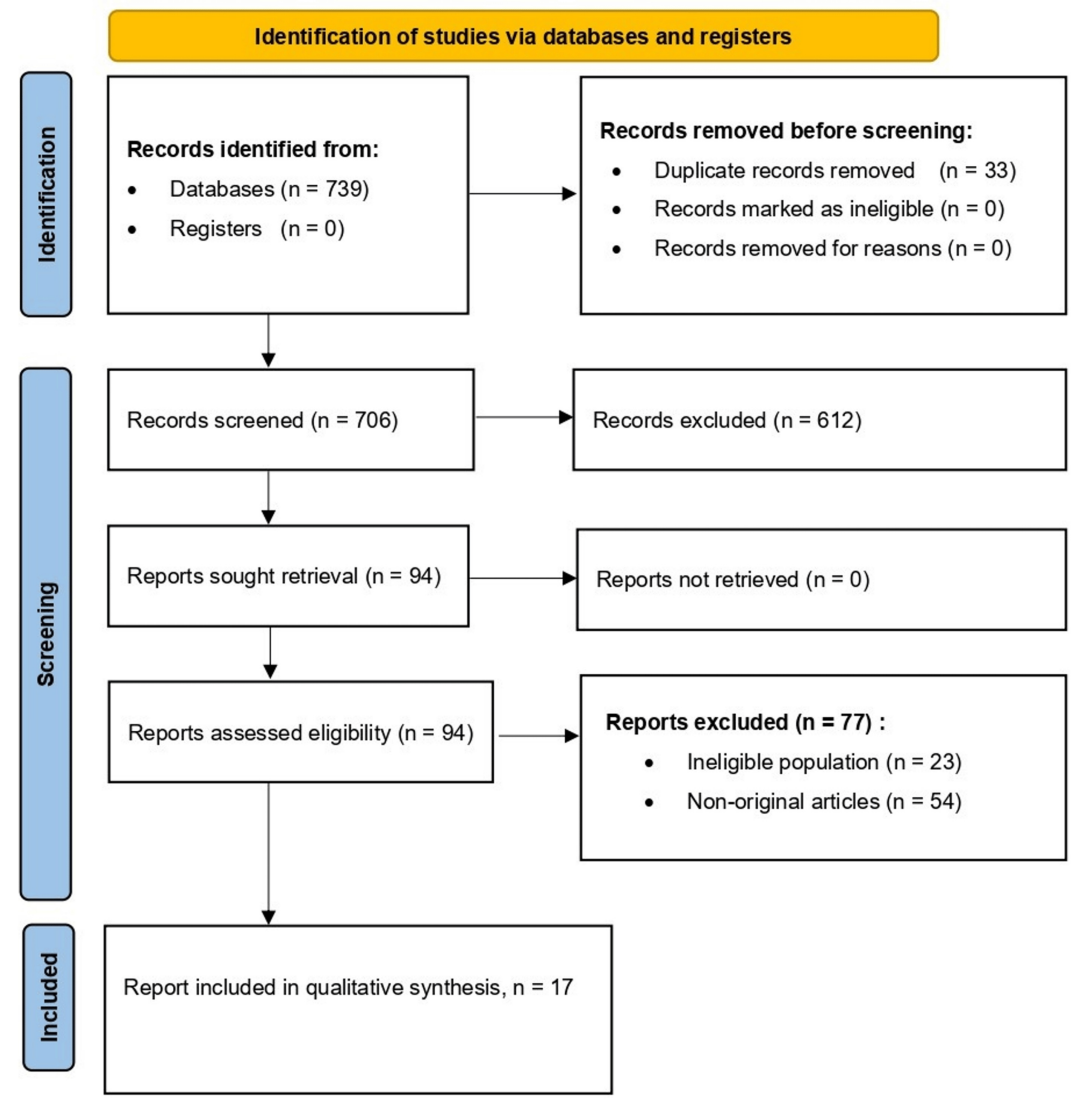
PRISMA flow diagram for study selection Records were identified through database searching, duplicates were removed before screening, and the remaining records were screened by title and abstract. Full-text reports were assessed for eligibility, with exclusions documented by reason. The final set of included studies represents reports included in the qualitative synthesis. PRISMA: Preferred Reporting Items for Systematic Reviews and Meta-Analyses

Records identified through database searching totaled 739. After duplicate removal, 706 records were screened by title and abstract, and 612 records were excluded. Ninety-four full-text articles were assessed for eligibility. Seventy-seven full-text articles were excluded for the following primary reasons: detection or classification only, not pre-seizure prediction (n = 21; 27.3%); no eligible scalp EEG data source (n = 14, 18.2%); review, survey, non-original, or insufficient publication type (n = 14, 18.2%); invasive or non-scalp EEG source not aligned with the review scope (n = 11, 14.3%); unclear or incompatible prediction task definition (n = 5, 6.5%); non-human or animal data (n = 4, 5.2%); unclear eligible EEG data source (n = 4, 5.2%); insufficient or unclear eligible sample size (n = 3, 3.9%); and unclear machine learning or deep learning approach (n = 1, 1.3%). Seventeen studies met the inclusion criteria and were included in the qualitative synthesis (Figure 1).

Study Characteristics and Methodological Design

Seventeen studies published between 2016 and 2025 were included. Most investigations relied on public datasets, most commonly CHB MIT (n = 13 of 17; 76.5%), with additional use of EPILEPSIAE (n = 3 of 17; 17.6%), AES Seizure Prediction Challenge data (n = 2 of 17; 11.8%), and the Siena scalp EEG dataset (n = 1 of 17; 5.9%). Because some studies used more than one dataset, dataset categories were not mutually exclusive. Two studies incorporated private datasets (n = 2 of 17; 11.8%). Although the review focused on scalp EEG, a subset evaluated mixed modalities or combined scalp EEG with intracranial EEG sources within the same study (n = 3 of 17; 17.6%). These mixed-modality studies were retained because they addressed seizure prediction or early warning. Still, their findings were interpreted with caution and not treated as direct scalp-only evidence when intracranial EEG results could not be separated.

Preictal definitions and operational prediction frameworks varied considerably, including fixed preictal windows, commonly 30 to 60 minutes, variable seizure occurrence periods explored by tuning, and inconsistent reporting of refractory periods and other clinically actionable warning constraints. Longer horizons, including windows up to approximately 60 minutes, were treated as methodological heterogeneity rather than as direct equivalents of the main 5- to 30-minute clinically focused window. Model families ranged from feature-based machine learning and synchronization metrics to deep learning, including CNNs, LSTMs, and GCNs, and several studies applied post-processing or calibration procedures intended to reduce false alarms. Deep learning models have frequently been reported to achieve high sensitivity in patient-specific settings; however, superiority over conventional machine learning could not be established because studies differed in their datasets, validation strategies, prediction horizons, post-processing, and false-alarm reporting. The key design and dataset characteristics of all included studies are summarized in Table 3.

**Table 3:** Included study characteristics, datasets, preictal definitions, features, models, and validation (N = 17) AES: American Epilepsy Society Seizure Prediction Challenge, CHB MIT: Children’s Hospital Boston Massachusetts Institute of Technology, CWT: continuous wavelet transform, DCGAN: deep convolutional generative adversarial network, EEG: electroencephalography, EMD: empirical mode decomposition, FAR: false alarm, FPR: false prediction rate, FIR: finite impulse response, GCN: graph convolutional network, iEEG: intracranial electroencephalography, LBP: local binary pattern, LDA: linear discriminant analysis, LOOCV: leave-one-out cross-validation, LSTM: long short-term memory, MODWT: maximal overlap discrete wavelet transform, NR: not reported, PLI: phase lag index, WPLI: weighted phase lag index, SNN: shallow neural network, SOP: seizure occurrence period, SPH: seizure prediction horizon, STFT: short time Fourier transform, SVM: support vector machine, TiW: time in warning

Study	Evidence group	Dataset or source	EEG type	N patients and seizures	Preictal window, SPH, or SOP	Model or feature approach	Validation strategy	Alarm reporting and interpretation note
Jia et al., 2022 [17]	Scalp public dataset	CHB MIT scalp EEG	Scalp	18 patients, seizures NR	60 min preictal window	Band energy, Hjorth parameters, GCN	LOOCV	False-alarm rate and time in warning were not reported, limiting clinical usability interpretation.
Segal et al., 2023 [18]	Scalp public dataset	Siena scalp EEG dataset, PhysioNet	Scalp	15 patients, 9 seizures	60 min to 30 s before onset	Base model outputs with risk controlling calibration	Synthetic validation and real data test	Explicitly focused on reducing false alarms, with sensitivity reduced after calibration.
Rasheed et al., 2021 [19]	Mixed scalp plus intracranial dataset	CHB MIT scalp plus Epilepsyecosystem intracranial	Scalp plus intracranial EEG	13 patients used, seizures NR	SPH 10 min, SOP 30 min	STFT spectrogram, DCGAN plus CNN, transfer learning	25% test split and k-fold cross-validation	Mixed EEG sources were interpreted cautiously and not treated as scalp-only evidence.
Costa et al., 2024 [20]	Scalp public dataset	EPILEPSIAE, Freiburg	Scalp	40 patients, 224 seizures	SOP 20 to 50 min, SPH 10 min	Linear features, logistic regression, SVM ensemble, SNN ensemble	Patient-specific, train the first 3 seizures and test the remaining	Alarm-based and forecasting perspectives were reported, supporting clinical usability interpretation.
Ozcan and Erturk, 2019 [21]	Scalp public dataset	CHB MIT public	Scalp	16 patients, 87 seizures	NR	Image-based EEG representation, 3D CNN	Train and test procedure NR	False prediction rate and warning time were reported, but validation details were limited.
Fathima et al., 2016 [22]	Scalp public dataset	CHB MIT public	Scalp	13 patients, 47 seizures	1 hour	One-dimensional local binary pattern histograms, LDA, SVM	Per patient seizure level, train and test	Performance varied by window size and post-processing settings.
Ibrahim et al., 2023 [23]	Mixed scalp plus intracranial dataset	CHB MIT plus AES Kaggle	Scalp plus intracranial EEG	23 or 24 subjects, 198 seizures or cases as reported	SPH 5 min, SOP 30 min	MODWT sub bands, multiresolution 1D CNN, post-processing	Patient-specific, hold out one seizure	Scalp and intracranial results were reported separately, supporting cautious interpretation.
Usman et al., 2017 [24]	Scalp public dataset	CHB MIT public	Scalp	24 patients, 84 seizures	Few minutes	EMD plus surrogate channel, statistical and spectral moments, SVM	Holdout is not fully explicit	Anticipation time was reported, but false-alarm reporting was incomplete.
Alkhrijah et al., 2025 [25]	Scalp public dataset	CHB MIT public	Scalp	24 patients, seizures NR	30 min	Feature fusion, ensemble classification, sequential minimal optimization	NR	Very high performance was reported, but split details and false-alarm rate units were not fully standardized.
Khan et al., 2018 [26]	Scalp private plus public dataset	MSSM private plus CHB MIT subset	Scalp	28 plus 22 patients, 68 seizure recordings	Transition about 10 min	CWT tensors and learned CNN features	Train and cross-validation, followed by an independent test	Independent testing strengthened validation, although selection criteria and post-processing influenced interpretation.
Aslam et al., 2022 [27]	Scalp public dataset	CHB MIT public	Scalp	22 subjects, seizures NR	Few minutes	STFT, statistical moments, CNN learned features, CNN plus LSTM	10-fold cross-validation	Hybrid handcrafted and learned features were used, but external validation was not reported.
Gao et al., 2022 [28]	Scalp public dataset	CHB MIT selected subset	Scalp	16 selected patients, seizures NR	SPH 5 min, SOP 30 min	Raw EEG, multi-scale CNN with dilated convolutions	LOOCV	Study excluded a 1 min intervention time and used patient-specific validation.
Batista et al., 2024 [29]	Scalp public dataset	EPILEPSIAE scalp EEG	Scalp	37 patients, 209 seizures	SOP explored, approximately 10 to 55 min; median about 20 min	Feature selection, SVM, post-processing chronology	LOOCV tuning, train, and test phases	Post-processing and explicit SPH or SOP handling improved the interpretability of alarm performance.
Detti et al., 2020 [30]	Scalp private dataset	Private new database	Scalp	14 patients, 47 seizures	T = 300 s or 900 s	PLI and WPLI synchronization features, patient-specific classifier	Leave one seizure out	Small private dataset and a high false-positive burden in some settings limited generalizability.
Andrade et al., 2024 [31]	Mixed multidatabase study	EPILEPSIAE plus CHB MIT plus AES plus Ecosystem	Scalp plus intracranial EEG	NR	SPH 10 min, SOP 10 to 55 min	Patient-specific machine learning pipeline	Mixed validation, chronological for scalp and 70/30 for intracranial data	Sample-based performance often appeared better than alarm-based performance, highlighting validation dependence.
Wu et al., 2023 [32]	Scalp public dataset	CHB MIT	Scalp	13 patients, 51 seizures	SPH 30 min, SOP 20 min	FIR filtering, STFT, LSTM	Patient-specific LOOCV	Alarm rule was specified, but validation remained patient-specific.
Ibrahim and Majzoub, 2017 [33]	Scalp public dataset	CHB MIT public	Scalp	Patients NR, 55 seizures	1-hour prediction horizon	Cross correlation and synchronization baselines, adaptive synchronization approach	Validation details NR	Designed for simplicity and low resource deployment, but validation reporting was limited.

Predictive Performance, False-Alarm Reporting, and Clinical Usability Metrics

Across the included studies, reported predictive performance was highly variable and often not directly comparable due to differences in validation strategies, datasets, preictal definitions, seizure-occurrence periods, seizure-prediction horizon configurations, post-processing rules, and alarm definitions. When sensitivity was explicitly reported, values ranged from 55% after calibration aimed at reducing false alarms to 100% in patient-specific settings, with many studies clustering in the high 80% to mid-90% range. Specificity was reported less consistently, ranging from 63% to 99.3%, where available. False-alarm metrics were inconsistently defined and underreported. Among studies that reported false alarms per hour, values ranged from 0.039 to approximately 0.73, with one synchronization-based evaluation reporting roughly two false positives per hour under certain settings.

Several studies addressed alarm burden through calibration or structured post-processing. However, high sensitivity alone was not considered sufficient evidence of clinical usability when accompanied by a substantial false-alarm burden, prolonged time-to-warning, unclear alarm definitions, or validation designs that did not reflect real-world deployment. Recurring limitations included dominance of patient-specific internal validation, limited external validation, incomplete patient-wise generalization, and inconsistent reporting of clinically actionable metrics such as time in warning and standardized alarm burden. Because performance metrics were defined and validated differently across studies, the values in Table 4 should be interpreted as descriptive study-level findings rather than pooled estimates or direct comparative rankings. Performance outcomes and false-alarm reporting formats are summarized in Table 4.

**Table 4:** Summary of predictive performance and false-alarm reporting across included studies Reported performance values were extracted as described in the original study and were not pooled due to heterogeneity across datasets, prediction windows, validation designs, and false-alarm definitions. AUROC: area under the receiver operating characteristic curve, FAR: false-alarm rate, FPR: false prediction rate, FP: false positives, LR: logistic regression, NR: not reported, SOP: seizure occurrence period, SPH: seizure prediction horizon, SS: skill score, SVM: support vector machine, TiW: time in warning

Study	Validation design	Horizon or lead time	Sensitivity or main performance	Specificity, AUROC, or accuracy	False alarm or time in warning reporting	Clinical usability interpretation
Jia et al., 2022 [17]	LOOCV	60 min preictal	Mean sensitivity 96.51%	Mean AUROC about 0.92	False-alarm rate and time in warning NR	High sensitivity was reported, but alarm burden was not available.
Segal et al., 2023 [18]	Synthetic validation and real EEG test	Preictal 60 min to 30 s	Sensitivity 100% to 55% after calibration	NR	False alarms reduced from 5.60 per hour to 0.45 per hour	Shows the trade-off between sensitivity and the reduced false-alarm burden.
Rasheed et al., 2021 [19]	25% test split and k-fold cross-validation	SPH 10 min, SOP 30 min	88.21% and 89.28%	NR	0.139 and 0.13 false predictions per hour	Mixed EEG sources limit direct scalp-only interpretation.
Costa et al., 2024 [20]	Patient-specific, train first 3 seizures	SPH 10 min, SOP 20 to 50 min	Prediction skill score 0.13 ± 0.26 for logistic regression	NR	Logistic regression 0.36 per hour, SVM 0.73 per hour, SNN 0.48 per hour; time in warning 0.19 ± 0.11 for logistic regression	Alarm-based and forecasting metrics improved clinical interpretation beyond what sensitivity alone could.
Ozcan and Erturk, 2019 [21]	Train and test procedure NR	NR	Sensitivity 85.7%	Accuracy 90.8%	0.096 false predictions per hour; time in warning 10.5 as reported	Validation reporting was limited, despite reporting the false-prediction rate and the time to warning.
Fathima et al., 2016 [22]	Per patient seizure level, train and test	Average prediction about 51.25 min	Sensitivity 100%	Specificity 95%	Average false prediction rate 0.463 as reported	Best performance depended on the selected window and post-processing settings.
Ibrahim et al., 2023 [23]	Patient-specific, hold out one seizure	SPH 5 min, SOP 30 min	CHB MIT sensitivity 82%	CHB MIT accuracy 85.114%	CHB MIT false prediction rate 0.058 as reported	Scalp and intracranial results were separated, but the scope of generalization remained limited.
Usman et al., 2017 [24]	Holdout not fully explicit	Average anticipation 23.48 min	Sensitivity 92.23%	Specificity 93.38%	False-alarm metric NR	Anticipation time was useful, but alarm burden was not reported.
Alkhrijah et al., 2025 [25]	NR	30 min preictal	Sensitivity 98.8%	Specificity 99.3%, AUROC 0.99	0.039 false alarms per hour as reported	Very high performance requires cautious interpretation because split details and units were not fully standardized.
Khan et al., 2018 [26]	Train and cross-validation, plus an independent test	10 min horizon	Sensitivity 87.8%	NR	0.142 false positives per hour	Independent testing improved robustness compared with purely internal validation.
Aslam et al., 2022 [27]	10-fold cross-validation	Average prediction 19.5 min	Sensitivity 93.8%	Specificity 91.2%, accuracy 94%	False-alarm metric NR	High accuracy was reported, but clinical usability was limited by the lack of an alarm burden.
Gao et al., 2022 [28]	LOOCV	SPH 5 min, SOP 30 min	Sensitivity 93.3%	NR	0.149 false prediction rate per hour	Patient-specific validation and false prediction reporting supported partial clinical interpretation.
Batista et al., 2024 [29]	LOOCV tuning and train-test phases	SPH 5 min, SOP about 20 min	Best seizure-related score 0.79	NR	Best false prediction rate about 0.05 per hour, as reported	Post-processing chronology helped clarify alarm behavior.
Detti et al., 2020 [30]	Leave one seizure out	T = 300 s or 900 s	NR	NR	About 2 false positives per hour at T = 900 s	High false positive burden under some settings limited clinical usability.
Andrade et al., 2024 [31]	Mixed, chronological for scalp and 70/30 for intracranial data	SPH 10 min, SOP 10 to 55 min	Variable	Variable, AUROC reported	False predictions per hour varied by database	Sample-based performance appeared more optimistic than alarm-based performance.
Wu et al., 2023 [32]	Patient-specific LOOCV	SPH 30 min, SOP 20 min	Mean sensitivity 86% as reported	NR	0.1189 per hour; time in warning 9.47%	Alarm rule was reported, but the evaluation remained patient-specific.
Ibrahim and Majzoub, 2017 [33]	Validation details NR	1-hour prediction horizon	84%, 46 of 55 seizures	Specificity 63%	False-alarm metric NR	Limited validation reporting reduced confidence in clinical translation.

Risk of Bias Within Included Studies

Overall risk-of-bias assessments indicated that no study achieved a low-risk overall judgment. Eight of 17 studies were rated as high risk of bias (47.1%), and nine were rated as unclear (52.9%). The most frequent drivers of high risk were weaknesses in analysis and validation, including incomplete independence between training and testing at the alarm or event level; potential information leakage from overlapping windows or unclear resampling; limited generalization evidence due to predominantly internal or patient-specific validation, and inconsistent reporting of clinically meaningful alarm metrics such as false-alarm rate and time in warning. Several studies were rated unclear rather than high because key methodological safeguards were insufficiently described, including explicit leakage prevention, event-level split logic, or alarm definition.

These risk-of-bias findings were interpreted as a central limitation of the evidence rather than merely a reporting issue. In particular, the frequent use of patient-specific internal validation and limited external validation made it difficult to determine whether high reported performance would persist in new patients, new datasets, or real-world monitoring conditions. Therefore, the performance findings were synthesized cautiously, with greater emphasis on validation design, alarm reporting, and clinical usability than on sensitivity alone. Domain-level judgments and the primary reasons for concerns are summarized in Table 5.

**Table 5:** Risk of bias summary across included studies, domain level judgments, and overall risk of bias FAR: false-alarm rate, LOOCV: leave-one-out cross-validation

Study	Domain 1: participants	Domain 2: predictors and EEG handling	Domain 3: outcome	Domain 4: analysis and validation	Overall	Short reason
Jia et al., 2022 [17]	Low	Unclear	Low	Unclear	Unclear	Leakage safeguards, false alarms, and clinical usability reporting are not fully described
Segal et al., 2023 [18]	Low	Unclear	Low	Unclear	Unclear	Split details and base model assumptions insufficient to fully rule out bias
Rasheed et al., 2021 [19]	Unclear	Unclear	Low	Unclear	Unclear	Cohort splitting and leakage controls not fully specified, mixed EEG sources
Costa et al., 2024 [20]	Low	Unclear	Low	Unclear	Unclear	Limited external validation and leakage detail
Ozcan and Erturk, 2019 [21]	Unclear	Unclear	Unclear	Unclear	Unclear	Validation independence and preprocessing details are insufficient for reporting
Fathima et al., 2016 [22]	Unclear	Unclear	Low	Unclear	Unclear	Window-level leakage controls and split logic are not fully described
Ibrahim et al., 2023 [23]	High	Unclear	Unclear	High	High	Mixed datasets and limited generalization, with incomplete outcome and validation details
Usman et al., 2017 [24]	Unclear	Unclear	Unclear	High	High	Split independence and false-alarm reporting unclear, generalization uncertain
Alkhrijah et al., 2025 [25]	Low	Unclear	Low	High	High	Validation split and false-alarm rate units are not fully standardized, risk of optimism
Khan et al., 2018 [26]	Low	Unclear	Low	Unclear	Unclear	Independent test helps, but leakage prevention details were limited
Aslam et al., 2022 [27]	Low	High	Low	Unclear	High	Overlap, oversampling, and unclear separation in cross-validation increase leakage risk
Gao et al., 2022 [28]	Low	Unclear	Low	Unclear	Unclear	Patient versus new patient generalization clarity is limited, and the false-alarm definition varies
Batista et al., 2024 [29]	Low	Unclear	Low	Unclear	Unclear	External validation is absent, and leakage safeguards are not fully explicit
Detti et al., 2020 [30]	High	Unclear	Unclear	High	High	Private small dataset, incomplete reporting, and no external validation
Andrade et al., 2024 [31]	High	Unclear	Unclear	High	High	Mixed datasets and heterogeneous validation and outcome definitions
Wu et al., 2023 [32]	Unclear	Unclear	Low	High	High	Patient-specific LOOCV only, subset selection, and possible optimistic bias
Ibrahim and Majzoub, 2017 [33]	Unclear	Unclear	Unclear	High	High	Validation design underreported and had limited detail on bias safeguards

Discussion

Summary of Principal Findings

Across the included evidence, the main finding was that EEG-based seizure prediction models can report moderate to high seizure sensitivity. Still, the clinical meaning of this performance depends strongly on how prediction is defined, validated, and translated into alarms. Studies using scalp EEG and public datasets commonly reported promising discrimination and sensitivity; however, the strength of this evidence was limited by heterogeneity in preictal definitions, seizure prediction horizons, seizure occurrence periods, post-processing rules, and validation designs. Therefore, the reviewed evidence supports technical feasibility more strongly than immediate clinical readiness.

A recurring pattern was the trade-off between seizure sensitivity and a clinically tolerable false-alarm burden. Jia et al. [17] reported high discrimination for a scalp EEG graph convolutional approach, with high mean sensitivity and strong area under the receiver operating characteristic curve (AUROC) performance under leave-one-out cross-validation, but false-alarm burden and time to warning were not reported. In contrast, Segal et al. [18] directly targeted alarm burden through risk-controlling calibration, showing that false alarms could be substantially reduced, although this reduction was accompanied by lower sensitivity. This pattern suggests that sensitivity should not be interpreted in isolation; it should be considered alongside specificity, calibration, false alarms per hour, time in warning, and the practical warning conditions under which the model operates.

Prediction Windows and Methodological Heterogeneity

Differences in model architecture contributed to variation across studies, but newer or more complex architectures should not be interpreted as inherently superior. Rasheed et al. [19] used generative synthesis and transfer learning, while Costa et al. (2024) [20] compared prediction and forecasting using conventional machine learning baselines in patient-specific settings. The apparent differences between these studies are likely influenced not only by model type but also by dataset composition, preictal labeling, seizure prediction horizon, seizure occurrence period, validation design, and alarm definition. For this reason, direct comparison between deep learning and conventional machine learning was limited. Therefore, comparative performance metrics across studies should be interpreted descriptively rather than as direct evidence that one model, dataset, or architecture is superior to another.

Input representation and feature strategy also influenced reported performance. Ozcan and Erturk [21] used an image-based 3D CNN approach and reported favorable accuracy with a low false-alarm rate. In contrast, Fathima et al. [22] used local binary pattern features with classical classifiers and reported high sensitivity and specificity but longer prediction times and a higher false-prediction rate. These findings indicate that earlier warning is not automatically more clinically useful. Longer warning horizons may provide more time for action, but they may also increase the time spent in warning or alarm if not carefully controlled. Therefore, comparisons between feature-based and end-to-end approaches require standardized reporting of seizure prediction horizon, seizure occurrence period, alarm definitions, and validation methods.

Several multicomponent pipelines combined preprocessing, feature extraction, classification, and post-processing. Ibrahim et al. [23] used multiresolution convolutional learning with wavelet-based decomposition and post-processing, whereas Usman et al. [24] used empirical mode decomposition-derived features and support vector machines. Complex pipelines may improve apparent discrimination, but incomplete reporting of alarm burden is a limitation of reporting and study design rather than a direct consequence of model complexity. Additionally, oversampling, overlapping windows, and unclear train-test separation may contribute to optimistic performance estimates if leakage safeguards are not fully described. These issues highlight the need to evaluate model performance in relation to both statistical discrimination and clinically interpretable alarm behavior.

Validation Design, Leakage Risk, and Alarm-Based Evaluation

The credibility of reported performance depended heavily on the validation design. Some studies reported very high headline metrics, including Alkhrijah et al. [25], whereas Khan et al. [26] included an independent test component and reported more conservative performance. This contrast supports a cautious interpretation: very high sensitivity or AUROC values are less informative when patient-wise separation, chronological testing, leakage prevention, and alarm definitions are unclear. Stronger evidence comes from studies that report both seizure-level sensitivity and alarm-based outcomes under validation conditions that better approximate future use.

The distinction between sample-based and alarm-based evaluation is central to interpreting seizure prediction studies. Sample-based evaluation classifies short EEG segments, whereas alarm-based evaluation assesses whether a system produces timely and tolerable warnings during continuous monitoring. Andrade et al. [31] highlighted that sample-based performance can appear more favorable than alarm-based performance because it may not fully reflect the temporal rarity of seizures or the burden of repeated warnings. Wu et al. [32] used an explicit patient-specific alarm rule and reported interpretable alarm-related operating characteristics. Time in warning is also clinically important because it reflects the proportion of monitoring time during which the patient or caregiver remains in warning. A model may correctly identify seizure risk but still be difficult to use if it keeps users in prolonged or frequent warning periods.

Post-processing and alarm logic appeared to be important components of clinically interpretable prediction systems. Batista et al. [29] emphasized post-processing as part of alarm generation, while Detti et al. [30] reported a higher false-positive burden in certain synchronization-based settings. Post-processing should therefore be reported as a reproducible component, including thresholds, refractory periods, decision rules, and alarm counting procedures. Without these details, it is difficult to determine whether performance gains reflect robust prediction, favorable datasets, or differences in alarm definitions.

Machine Learning, Deep Learning, and Clinical Translation

The included studies suggest that both conventional machine learning and deep learning approaches can produce promising results. Still, the comparative superiority of deep learning over conventional machine learning could not be established. Deep learning models, including CNNs, LSTMs, GCNs, and multiresolution architectures, have frequently demonstrated high performance in patient-specific settings. However, these findings were often limited by small sample sizes, heterogeneous datasets, internal validation, variable prediction windows, and inconsistent alarm reporting. Thus, the review supports cautious interpretation of deep learning performance rather than a conclusion that architectural complexity alone improves clinical applicability.

The current evidence base should be viewed as early-stage or proof-of-concept literature rather than evidence of readiness for routine patient application. Many studies aimed to develop algorithms, test feasibility, or compare modeling strategies rather than evaluate deployable clinical systems. This distinction is important because clinical seizure warning systems require more than high sensitivity. They require tolerable false-alarm rates, acceptable warning time, clear calibration, robustness to changing EEG conditions, and validation on patients or datasets not used during model development.

The distinction between detection, prediction, and forecasting also remains important. Detection identifies an ongoing or already occurring seizure, prediction aims to warn before an imminent seizure, and forecasting estimates a broader period of increased seizure risk. Although these concepts all involve seizure-related EEG classification or risk estimation, they represent different clinical tasks and should not be interpreted as equivalent. Clear terminology is necessary so that readers can understand whether a model is intended to identify seizures as they occur, provide an actionable early warning, or estimate longer-term seizure risk.

Future Directions

Future seizure prediction studies should include stronger safeguards against optimistic performance estimates. These safeguards include patient-wise or chronological data splitting, strict separation between training and testing data, avoidance of overlapping window leakage, predefined refractory alarm periods, transparent alarm smoothing rules, standardized definitions of the seizure prediction horizon and seizure occurrence period, and consistent reporting of false alarms per hour and time in warning.

Study Limitations

Interpretation of comparative performance is limited by differences across studies in prediction horizons, seizure occurrence periods, datasets, model types, post-processing rules, and validation methods, thereby reducing direct comparability. Several studies did not consistently report clinically relevant alarm outcomes, including false-alarm burden, calibration, and time spent under warning. External or prospective validation was limited, and methodological reporting was sometimes insufficient to fully rule out optimistic performance estimates related to patient-specific validation, overlapping windows, or unclear train-test separation.

This review also has limitations. Only 17 studies were included in the qualitative synthesis, which limits the strength of broad conclusions about EEG-based seizure prediction. The restriction to English language records may have excluded relevant non-English studies. Publication bias may occur because studies with favorable model performance are more likely to be published. The protocol was not prospectively registered, which should be considered when interpreting the review process. A meta-analysis was not performed because quantitative pooling was not appropriate given the heterogeneity across datasets, prediction windows, validation designs, model architectures, and false-alarm definitions. Incomplete reporting across the included studies, particularly for false-alarm metrics, time in warning, calibration, and external validation, further limited the certainty of the evidence.

## Conclusions

The included studies suggest that scalp EEG-based seizure prediction has technical feasibility and research promise, but the current evidence does not establish readiness for routine clinical implementation. Reported performance remains limited by heterogeneous prediction windows, inconsistent false-alarm reporting, patient-specific validation, small samples, and limited external or prospective testing.

Deep learning models have often been reported to perform strongly, but superiority over conventional machine learning could not be confirmed due to major methodological differences across studies. Future research should prioritize standardized alarm-based outcomes, leakage-resistant validation, external testing, and prospective evaluation before EEG-based seizure prediction can be considered clinically reliable.
